# Effect of Valve Height on the Opening and Closing Performance of the Aortic Valve Under Aortic Root Dilatation

**DOI:** 10.3389/fphys.2021.697502

**Published:** 2021-08-30

**Authors:** Qianwen Hou, Guimei Liu, Ning Liu, Honghui Zhang, Zhuoran Qu, Hanbing Zhang, Hui Li, Youlian Pan, Aike Qiao

**Affiliations:** ^1^Faculty of Environment and Life, Beijing University of Technology, Beijing, China; ^2^Intelligent Physiological Measurement and Clinical Translation, Beijing International Base for Scientific and Technological Cooperation, Beijing, China

**Keywords:** aortic valve, aortic root dilatation, aortic valve repair, biomechanics, numerical simulation

## Abstract

Patients with aortic valve disease can suffer from valve insufficiency after valve repair surgery due to aortic root dilatation. The paper investigates the effect of valve height (Hv) on the aortic valve opening and closing in order to select the appropriate range of Hv for smoother blood flow through the aortic valve and valve closure completely in the case of continuous aortic root dilatation. A total of 20 parameterized three-dimensional models of the aortic root were constructed following clinical surgical guidance. Aortic annulus diameter (DAA) was separately set to 26, 27, 28, 29, and 30 mm to simulate aortic root dilatation. H_V_ value was separately set to 13.5, 14, 14.5, and 15 mm to simulate aortic valve alterations in surgery. Time-varying pressure loads were applied to the valve, vessel wall of the ascending aorta, and left ventricle. Then, finite element analysis software was employed to simulate the movement and mechanics of the aortic root. The feasible design range of the valve size was evaluated using maximum stress, geometric orifice area (GOA), and leaflet contact force. The results show that the valve was incompletely closed when H_V_ was 13.5 mm and D_AA_ was 29 or 30 mm. The GOA of the valve was small when H_V_ was 15 mm and D_AA_ was 26 or 27 mm. The corresponding values of the other models were within the normal range. Compared with the model with an H_V_ of 14 mm, the model with an H_V_ of 14.5 mm could effectively reduce maximum stress and had relatively larger GOA and less change in contact force. As a result, valve height affects the performance of aortic valve opening and closing. Smaller H_V_ is adapted to smaller D_AA_ and vice versa. When H_V_ is 14.5 mm, the valve is well adapted to the dilatation of the aortic root to enhance repair durability. Therefore, more attention should be paid to H_V_ in surgical planning.

## Introduction

The aortic root consists of the sinuses of Valsalva, aortic valve, aortic annulus (AA), aorto-ventricular junction, and sinotubular junction (David, 2013). The aortic valve controls unidirectional blood flow from the left ventricle to the aorta by performing a regular opening and closing movement with contraction and relaxation of the heart. Aortic valve insufficiency (AI) and aortic stenosis (AS) represent the most common aortic valve diseases (Alkhodari and Fraiwan, 2021; Wazaren et al., 2021; Zhang et al., 2021). AI causes the blood to flow back into the left ventricle, leading to left ventricular dysfunction and even diastolic heart failure. As for AS, patients with moderate to severe AS can develop obstruction of the left ventricular output and reduction in cardiac output, which may cause myocardial insufficiency, angina pectoris, and even sudden death. Therefore, it is essential to guarantee a normal function of the aortic valve.

For young patients who suffer from aortic valve dysfunction, it is challenging to decide on the choice of surgical procedure. For example, mechanical valve replacement typically necessitates lifelong anticoagulant therapy, which increases the risk of hemorrhage and thromboembolism and decreases the quality of life of patients. On the other hand, biologic prosthetic valves have a higher risk of reoperation compared with mechanical valves due to lack of structural durability, which leads to a significant increase in mortality (Natalie et al., 2016; Goldstone et al., 2017). In the past two decades, aortic valve preservation or repair operations have become increasingly popular alternatives to aortic valve replacement for tricuspid and bicuspid valves. In young patients, valve repair is more likely to provide better quality of life and fewer valve-related complications compared with prosthetic valve; however, this has to be balanced against the risk of reoperation (Schäfers et al., 2013; Arabkhani et al., 2015; Lansac et al., 2015; Emmanuel et al., 2016; Ravil et al., 2019).

In clinical practice, gradual expansion and deformation of the aorto-ventricular junction have been observed as the age of patients increase, resulting in an increase in aortic annulus diameter (D_AA_). An untreated dilated aortic annulus of more than 25–28 mm can result in aortic regurgitation (AR) or AS, representing a major risk factor for the failure of aortic valve repair operations (Aicher et al., 2012; Navarra et al., 2013; Laurent et al., 2014). In such cases, reoperation is required to restore the normal occlusion of the aortic valve. Therefore, before the initial repair operation in young patients, physicians should consider that the dilatation of the aortic root can cause reappearance of the valve dysfunction and limit the durability of the repair to a few years after the operation. Hence, determining the geometric size design of the aortic valve to adapt to aortic root dilatation and enhance repair durability is a key challenge.

As a functional unit, the geometric interrelation between the aortic valve and root has led to realizing that the reconstruction of near-normal valve and root geometry is essential to obtain a good functional repairing result. For surgeons, it is necessary to design a specific valve according to requirements when functional dimensions are restored in the leaky valve of a patient (Pan et al., 2014). Previous studies have been done on the influence of aortic root geometry on valve closure performance to evaluatethe aortic valve sparing surgery before the operation, since the numerical models feature numerous advantages over the attempts of surgeons. Further studies (Gnyaneshwar et al., 2002; Howard et al., 2003; Weltert et al., 2013) constructed finite element models to perform simulation studies, and their results revealed that the closing performance of the aortic valve could be affected by increase in the size of the aortic root. Besides, they reported that the expansion of the aortic root was the main factor leading to increased pressure of the leaflets and then to AI. By numerical simulation, Marom and colleagues formulated six idealized models of the aortic root separately with a D_AA_ of 20 and 30 mm. The results proved that the changes in D_AA_ produced a significant inhibitory effect on aortic valve performance (Marom et al., 2013). Additional simulation results indicated that increasing leaflet graft height leads to an increase in the amount of growth that the reconstructed valve can accommodate. Furthermore, for a given vessel size, an increased valve height (H_V_) leads to better coaptation metrics (Hammer et al., 2016). We have recently performed studies on the aortic root by building simulation models with or without fluid-structure interactions (Pan et al., 2014, 2015; Qiao et al., 2014; Li et al., 2019). The above-mentioned studies identified the factors of H_V_ and effective height (EH) as important parameters to determine the acute and long-term functions of repaired aortic valves. However, the valve repair procedure has not yet resulted in good functional stability, which indicates that this pathologic entity requires a specific approach. Thus, determining the suitable H_V_ and reconstructing the aortic valve to adapt to the dilatation of the root at the time of the initial repair can have important prognostic implications for repair durability, which can achieve the best stabilization effect.

In this study, 20 groups of finite element models were established to simulate the process of valve opening and closing by numerical simulation. Then, the maximum stress, leaflet contact pressure, and effective orifice area (GOA) in different models were compared to evaluate valve performance. Therefore, the feasible range of the valve size in the case of continuous dilatation of the aortic root can be obtained. The obtained data might serve as basis for decision making in aortic valve repair procedures.

## Methods and Materials

### Three-Dimensional Geometric Modeling

**Figure 1:**
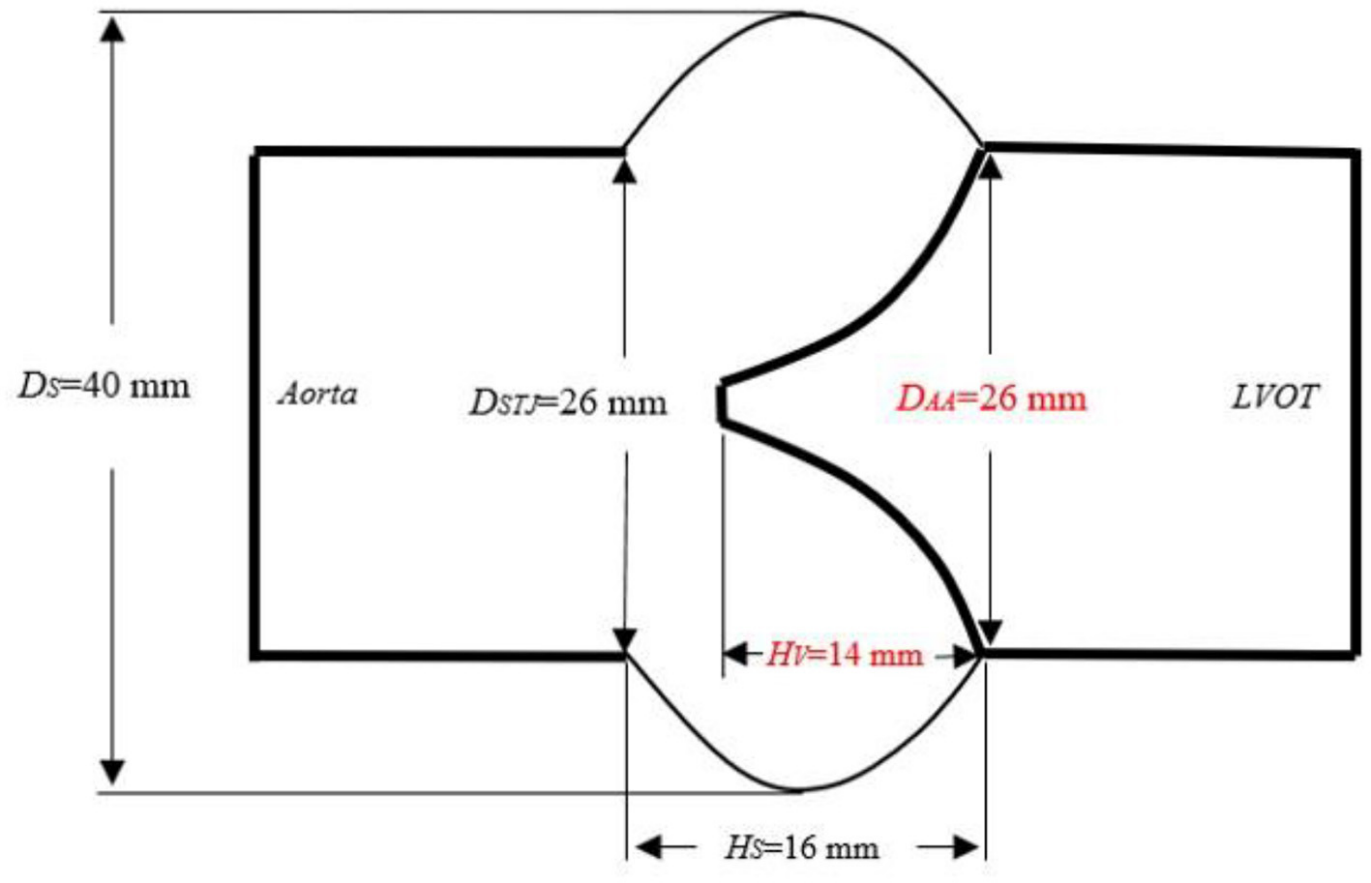
Schematic diagram of the aortic root size. D_AA_, aortic annulus diameter; H_V_, valve height; L_H_, valve length; D_STJ_, diameter of the sinotubular junction; H_s_, sinus height; D_S_, sinus diameter; LVOT, left ventricular outflow tract.

The three-dimensional geometry of a base aortic valve was constructed using geometric constraints and modeling dimensions retrieved from literature and the clinical surgical guidance as the reference model (aortic annulus diameter, D_AA_ = 26 mm; sinotubular junction diameters, D_STJ_ = 26 mm; sinus diameter, D_S_ = 40 mm, valve height, H_V_ = 14 mm; sinus height, H_S_ = 16 mm). The left and right coronary arteries connected with the aortic sinus were removed (Figure 1) (Labrosse et al., 2011; Pan et al., 2015; Hou et al., 2019). On this basis, we optimized the geometrical structure of the leaflet and aortic sinus, and proposed a new model of the aortic root closely similar to its physiological structure.

**Figure 2:**
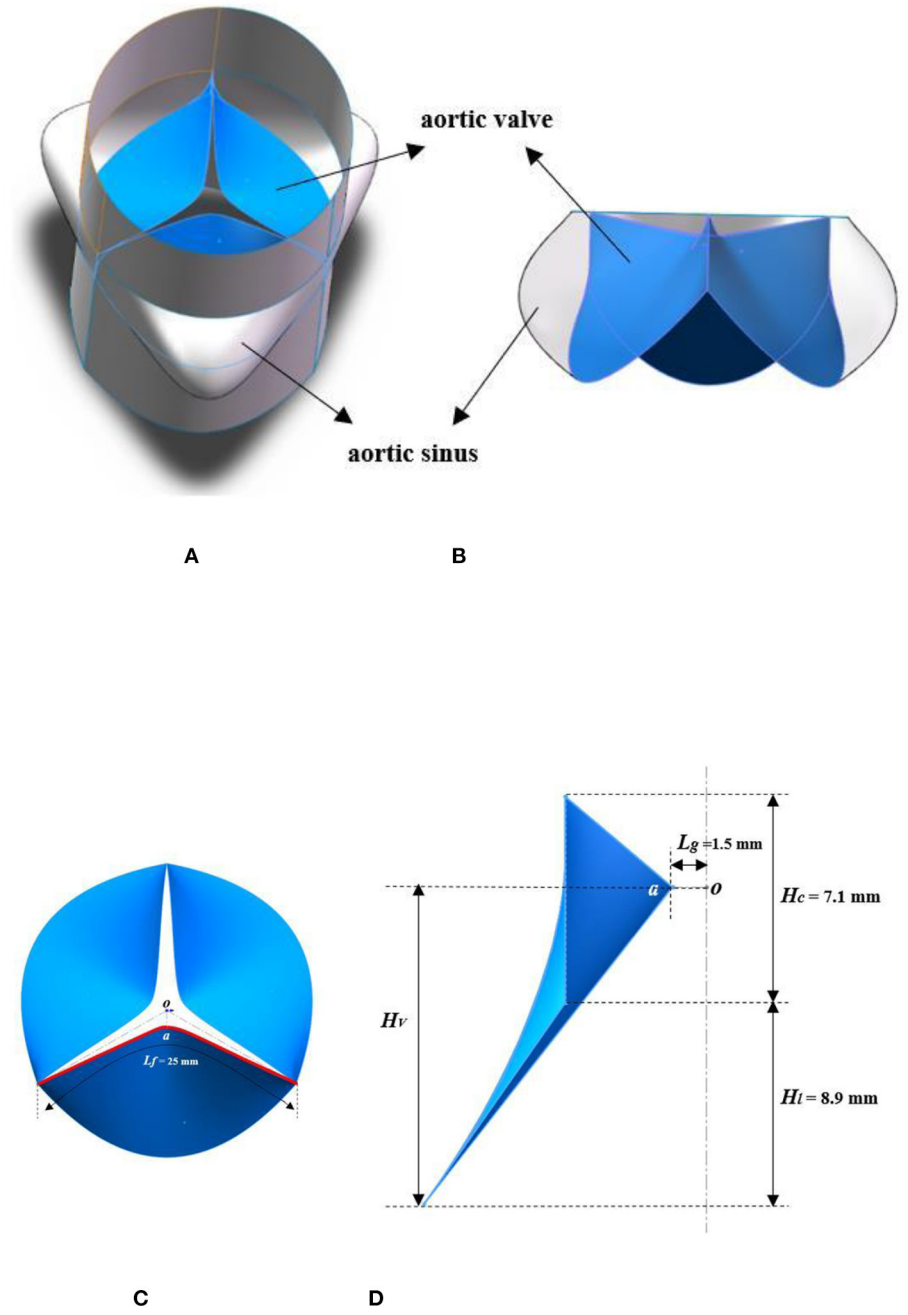
Schematic diagram of the 3D geometry models. **(A)** 3D structure model of the aortic root; **(B)** structure of the aortic sinus and leaflet; **(C)** structural feature of the aortic valve; **(D)** dimensional characteristics of leaflet. H_V_, valve height; H_l_, leaflet height; H_c_, commissure height; L_g_, gap length; L_f_, free-edge length.

First, the contour of the aortic sinus and leaflet was constructed using a series of arcs with different heights implemented in the SolidWorks 2015 software (Solidworks, Waltham, MA, United States), and the model with the geometric characteristics of the aortic sinus and leaflet was obtained by filling the curved surfaces. In order to conveniently constrain the variables in this study, three aortic sinuses and leaflets were assumed to be uniform and symmetrical, and used to obtain the shell structure of the aorta by putting them in an array with the center axis of the valve annulus as the rotation axis with an interval of 120° (Figure 2B). Then, in order to ensure full blood flow in the aortic root and restore the real structure of the model to the greatest extent, two straight-tube extensions (length: 20 mm) were added to the inlet (ventricular extension) and outlet sections (aortic extension) of the aortic root to enhance computational stability (Figure 2A) (Cao and Sucosky, 2015; Pan et al., 2015; Li et al., 2019). In all the models, the reference state consisted of the valve in a partially-open configuration (i.e., in transition between the coapted state and the opening state) and was constructed by leaving a small gap (Lg = 1.5 mm) between the free edge (Lf = 25 mm) of the leaflets (Figure 2C) (Cao et al., 2016). The dimensions of the aortic valve are reported in Figure 2D (Labrosse et al., 2010; Li et al., 2019).

Based on the reference model, valve height was maintained unchanged, and aortic annulus diameter was increased first. D_AA_ was separately set to 26, 27, 28, 29, and 30 mm to simulate the dilatation of the aortic root. Then, H_V_ was separately set to 13.5, 14, 14.5, and 15 mm to simulate aortic valve alterations in surgery. During the establishment of the models, other parameters remained unchanged, and H_V_ and D_AA_ were modified to simulate the movement and mechanics of the aortic valve under aortic root dilatation conditions. Overall, a total of 20 parameterized 3D finite element models of the aortic root were constructed.

### Meshing Generation and Element Settings

All the three-dimensional models were imported into the HyperMesh 13.0 software (HyperMesh, Altair, United States) to accomplish mainly mesh generation, which included the definition of the nodes at the upper and lower ends of the aortic root and elements in different parts of the root (three leaflets, the vessel wall of the aorta, the sinus and the left ventricle outflow tract). We referred to the mesh elements that are commonly used in the existing finite element analysis of the aortic valve (Oomen et al., 2018), such that the aortic root model was divided into shell elements. The model consisted of two parts: the aortic valve and aortic wall. The shape of the artery wall was regular, which was divided into rectangular mesh according to mesh dependence analysis. Since this study mainly focused on simulation results of the sutured and free edges of the aortic valve, the meshes of these parts were all divided into neat triangular elements in order to smooth the deformed surface and determine the stress–strain relationship of the aortic valve as accurate as possible.

**Figure 3:**
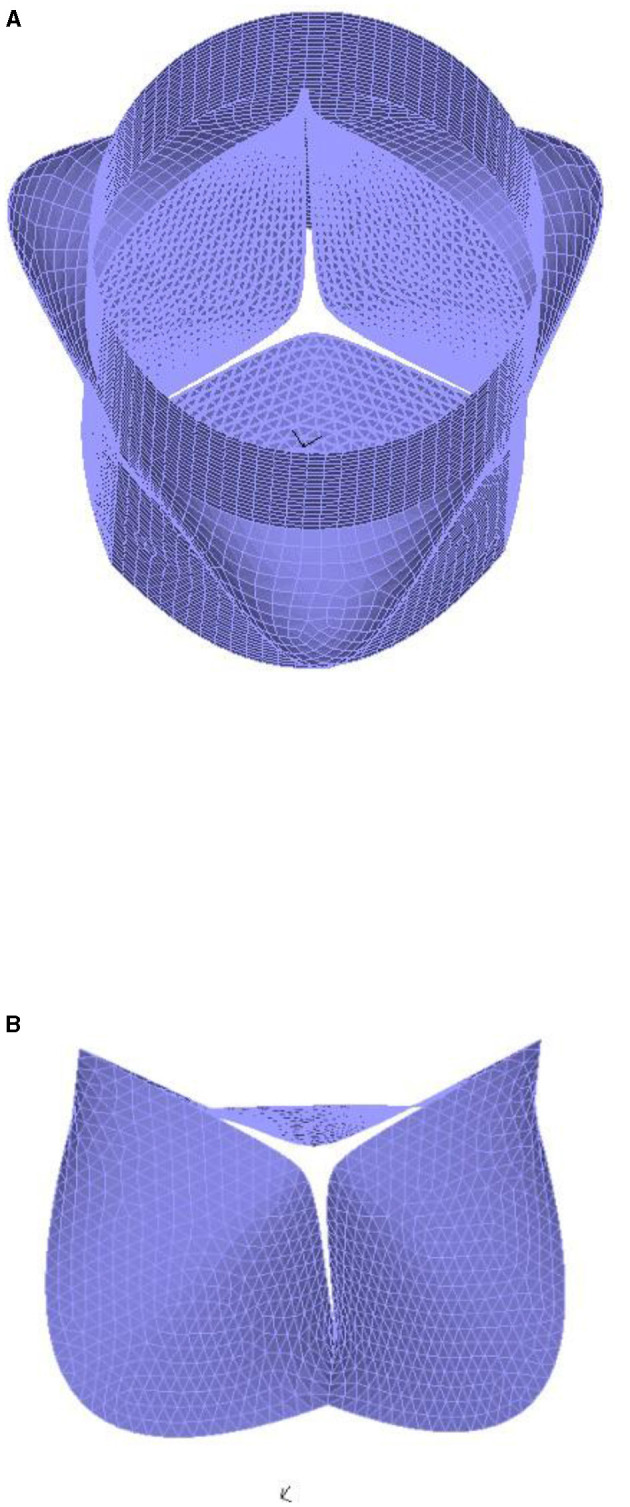
The mesh of the aortic root. **(A)** the whole model; **(B)** the leaflets.

A mesh sensitivity analysis was performed based on the maximum stress value over the aortic valve to determine an appropriate mesh density for the model and ensure a numerical convergence. This analysis was conducted on the initial geometric model. Refining the mesh (e.g., with an element size a total of 0.4 mm and consisted of 7,022 elements) did not affect the stress by <5%, which was considered sufficiently resolved to capture the valvular dynamics. Therefore, this structure mesh was employed in the remaining simulations. Figure 3 shows the mesh of the overall model and the leaflets. Finally, the model was imported into the finite element software to perform varied operations, such as constraints, loading, and parameter settings.

### Numerical Simulation of the Aortic Root

#### Material Properties

In this study, the material properties and boundary conditions were set using the finite element software ADIAN 9.3 (ADINA, Watertown, MA, United States), and the numerical simulations of the structural mechanics were completed. The shell element was selected as the element type of the valve and vessel wall. Physiologically speaking, the aortic valve tissue exhibits an obvious fiber arrangement (Feng et al., 2020), which belongs to a hyperelastic and anisotropic material. In fact, during systole, valve leaflets typically experience strains below 10% (Weinberg and Mofrad, 2007; Cao and Sucosky, 2017) and essentially behave as an isotropic material (Billiar and Sacks, 2000). The progressive locking of the collagen fibers increases material stiffness along the circumferential direction during diastole, and the valve is mostly or fully closed during this phase, which results in negligible leaflet stress levels. Therefore, the anisotropy of the leaflet material could potentially alter leaflet curvature during coaptation; it is expected to have a slow impact on regional leaflet stress. Moreover, the pressure gradient between the ventricle and the aorta is in the range of 0–14 kPa, and the stress-strain relationship of the valve leaflet is linear in this range. Therefore, the isotropic and linear elastic material properties matching the element type were assumed in the present models so as to simplify the computation and improve the feasibility of the analysis (Auricchio et al., 2014; Qiao et al., 2014; Hammer et al., 2016; Marom et al., 2016). This assumption is feasible to simulate the coaptation and avoid the problem of excessive distortion of the mesh during the contact process of the leaflets. Young's modulus of 1 and 2 MPa, densities of 1,100 and 2,000 kg/m^3^, and thicknesses of 0.3 and 0.6 mm were used for the valve and the rest of the aortic root, respectively (Marom et al., 2012; Rim et al., 2014; Hou et al., 2019). Poisson's ratio used was 0.45 (Katayama et al., 2008; Pan et al., 2015).

#### Boundary Conditions

**Figure 4:**
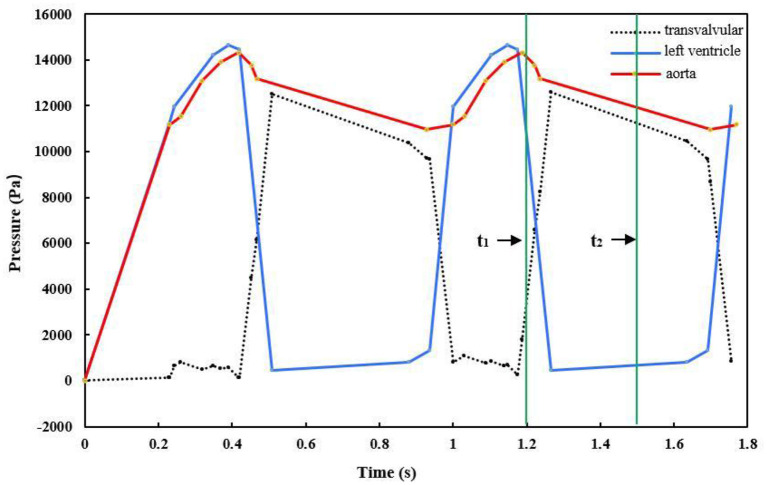
Pressure waves exerted on the aorta, left ventricle, and leaflets (t1 = 1.2 s, t2 = 1.5 s).

First, the nodes at the aortic outlet and ventricular outflow were fixed in all directions, with zero degrees of freedom to prevent deflection. Then, time-dependent physiological pressure conditions (Figure 4) were applied to the aorta, valve, and left ventricle. The difference between the pressure values on the side of the left ventricle and side of the aorta represented the transvalvular pressure gradient, which drives the aortic valve to move periodically (Pan et al., 2015; Li et al., 2019). In the simulation, two complete continuous pressure differences were loaded on the aortic valve. Finally, since the analysis started from the unpressurized geometry, a solution phase of 0–0.2 s before the systole was added to the simulation for the transition of the model from the zero-stress state to normal physiological pressure. Thus, the initial state of the model was consistent with the loading conditions at the end of the diastole, and calculation accuracy was improved (Labrosse et al., 2011; Pan et al., 2015).

#### Computational Method and Control Parameter

In the ADINA 9.3 (ADINA, Watertown, MA, United States) software, the implicit dynamics method based on displacement parameters was chosen to solve the partial differential equations of the structural analysis. The iteration method used was Modified Newton. In practice, in energy dissipation, the vibration response of the structure would gradually weaken without external force. The complex energy dissipation mechanism is expressed by damping, so we set the damping coefficient of the aortic valve to 0.15 to promote convergence (Weltert et al., 2013). When the aortic valve is closed, the three leaflets contact each other, and the contact type is defined as the friction contact; we set the friction coefficient to 0.01. The time step was set to 0.001 or 0.0001 in phases where the pressure load changed slowly or drastically, respectively.

All the models were calculated using the finite element software ADINA9.3. Since periodic dependence exists in finite element analysis, the research group carried out a study on periodic issues by gradually increasing the number of cycles. The results showed that two cycles already had a good convergence effect (Pan et al., 2015). The effect of increasing the cycle did not change after two cycles, and the error of the adjacent cycles was <5%. Hence, we selected two cardiac cycles to obtain convergence results to save computation time.

## Results

**Figure 5:**
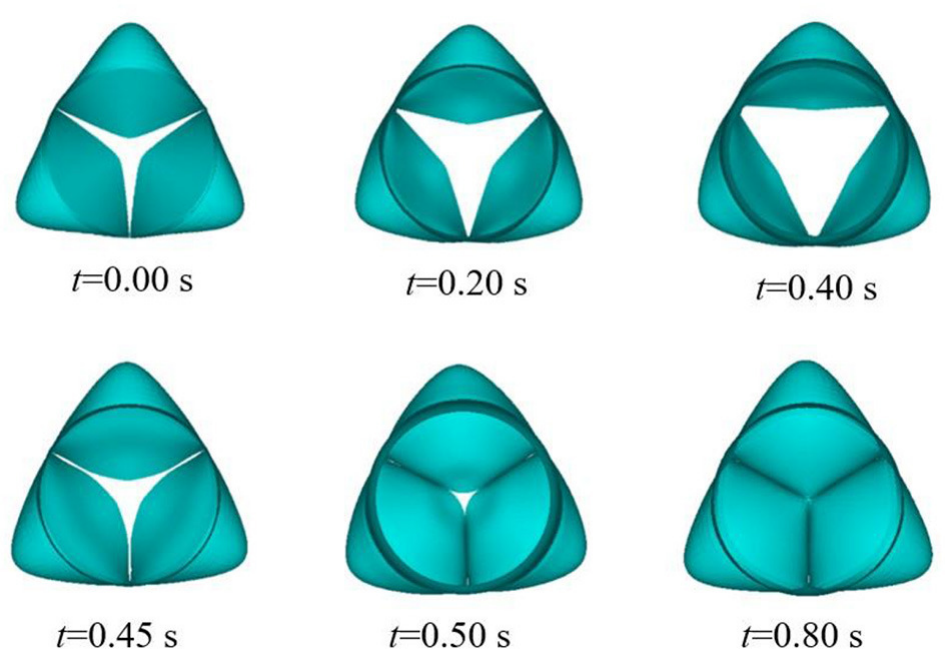
Aortic valve opening and closing process in finite element numerical simulation.

We simulated the dynamics of the aortic root over two cardiac cycles. During the period of 0–0.2 s, the pressure was prescribed as an initial pressurization phase. Then, the period of 0.2–0.4 s included rapid ejection of blood from the left ventricle into the aorta, because the left ventricular pressure exceeded the pressure within the aorta, and the aortic valve opened driven by the transvalvular pressure. During the period of 0.4–0.7 s, the left ventricular pressure began to decrease to a certain value, and the aortic pressure forced the aortic valve to close. The aortic valve remained closed for a period of time, then the next cardiac cycle began. Figure 5 shows the movement of the aortic valve during a cardiac cycle in the reference model. The calculation results from the second cardiac cycle were used for the analysis. The maximum GOA of the valve was extracted at time t_1_ = 1.2 s when the aortic valve was fully opened, while the values of the maximum stress and leaflet contact force were obtained at time t_2_ = 1.5 s when the valve was completely closed. In the following, we present the numerical simulation results according to the four cases of the valve height.

### Setting H_V_ to 13.5 mm

The mechanical parameters of the aortic valve with an HV of 13.5 mm are shown in Table 1. The maximum stress value appeared to increase as the D_AA_ increased. The contact force of the leaflets presented a decreasing trend, while the GOA of the aortic valve presented an increasing trend when the parameter D_AA_ increased from 26 to 30 mm.

**Table 1:** Parameter values of the model with a valve height of 13.5 mm.

**Parameter/D_**AA**_ (mm)**	**26**	**27**	**28**	**29**	**30**
Maximum stress (kPa)	676	711	788	859	911
Contact force (N)	6.32	4.6	3.11	1.56	0.53
GOA (mm^2^)	200.10	211.33	216.05	220.10	230.67

**Figure 6:**
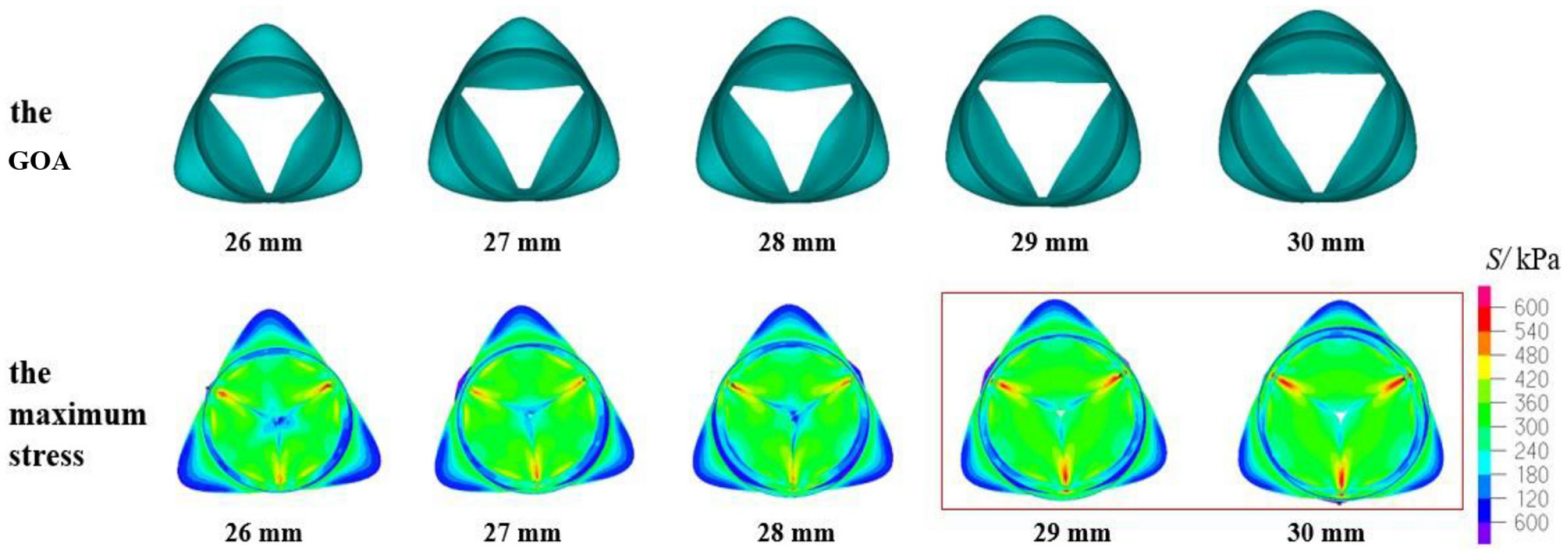
Geometric orifice area (GOA) of the valve and maximum stress distribution of the model with valve height = 13.5 mm and aortic annulus diameter of 26–30 mm.

The geometric orifice area and the maximum stress distribution of the models with a valve height of 13.5 mm and aortic annulus diameter of 26–30 mm are shown in Figure 6. It can be found that the maximum stress was in accordance with what was reported in previous studies (Labrosse et al., 2011; Marom et al., 2013) when the D_AA_ was 26, 27, or 28 mm. Meanwhile, the contact force of the leaflets was close to the results of 5.43 N in the study of Qiao et al. (2014). The orifice area of the valve was >200 mm^2^, which can meet the requirement of the clinical standard. When the D_AA_ was 29 or 30 mm, the GOA was within the normal range, and the contact force was slightly smaller. Larger stress occurred at the junction of the leaflets and sinus, and there were several incomplete closures of the leaflet joints, while the maximum stress and contact force were relatively small. The reason may be the large insufficiency of the valve at 1.5 s of the second cardiac cycle.

### Setting H_V_ to 14 mm

The mechanical parameters of the aortic valve with a valve height of 14 mm are shown in Table 2. The GOA of the aortic valve showed an increasing trend, while the contact force of the leaflets tended to decrease as the D_AA_ increased from 26 to 30 mm.

**Table 2:** Parameter values of the model with a valve height of 14 mm.

**Parameter/D_**AA**_ (mm)**	**26**	**27**	**28**	**29**	**30**
Maximum stress (kPa)	629	757	878	864	893
Contact force (N)	7.09	5.52	4.22	3.47	3.10
GOA (mm^2^)	209.10	209.75	210.11	216.34	230.67

**Figure 7:**
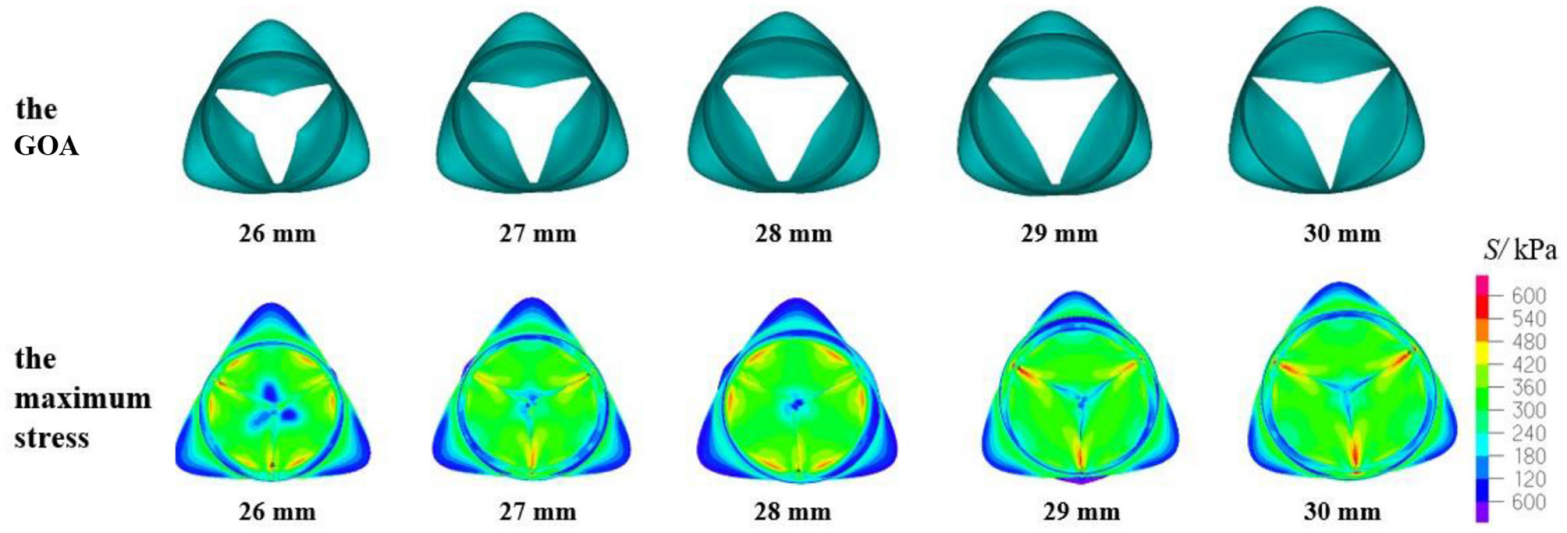
GOA of the valve and maximum stress distribution of the model with H_V_ = 14 mm and D_AA_ of 26–30 mm.

The geometric orifice area and maximum stress distribution of the models with a valve height of 14 mm and aortic annulus diameter of 26–30 mm are shown in Figure 7. When the D_AA_ was 26, 27, or 28 mm, the maximum stress and the contact force of the leaflets were similar to the previously reported simulation results (Howard et al., 2003; Marom et al., 2013; Weltert et al., 2013). The GOA met the clinical standard. When the D_AA_ was 29 or 30 mm, the GOA was within the normal range, but the maximum stress values were >800 kPa and greater stress also occurred at the junction of the leaflets and sinus. On the other hand, the contact force was relatively small, similar to the model with an H_V_ of 13.5 mm, which represents the major risk factor for AI.

### Setting H_V_ to 14.5 mm

**Figure 8:**
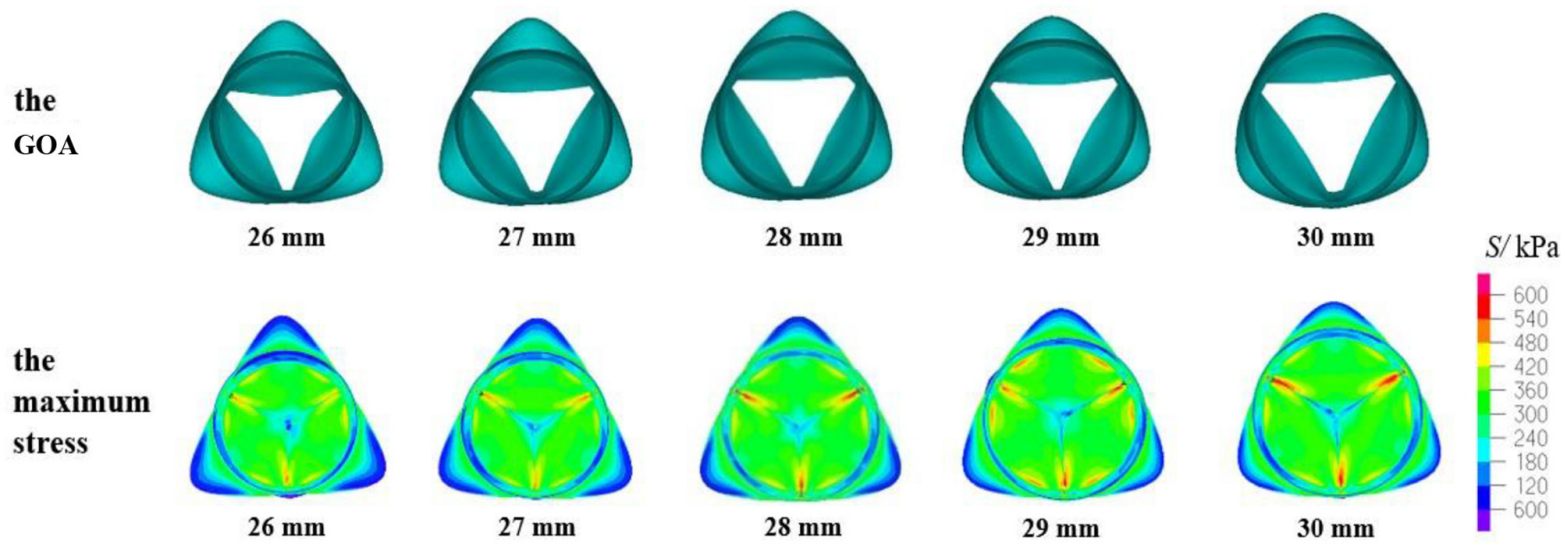
GOA of the valve and maximum stress distribution of the model with H_V_ = 14.5 mm and D_AA_ of 26–30 mm.

**Figure 9:**
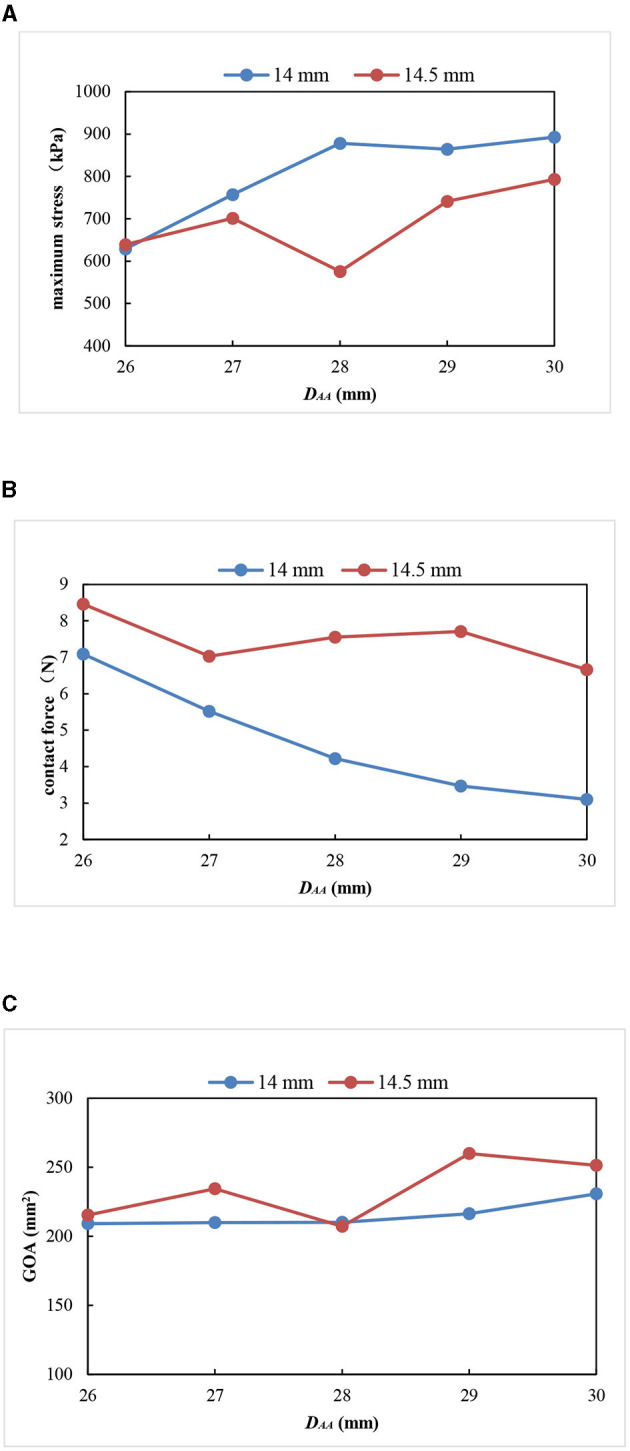
Comparison of three parameters between the models with an H_V_ of 14 and 14.5 mm. **(A)** Maximum stress; **(B)** contact force; **(C)** geometric orifice area.

The mechanical parameters of the aortic valve with a valve height of 14.5 mm are shown in Table 3. When the H_V_ was 14.5 mm, the three evaluation parameters affecting the opening and closing performance of the aortic valve were within a reasonable range, and the aortic valve of all the models could achieve complete closure and opening. Figure 8 shows the GOA and maximum stress distribution of the models with an H_V_ of 14.5 mm and D_AA_ of 26–30 mm. A comparison of the three parameters between the models with an H_V_ of 14 and 14.5 mm, respectively, is shown in Figure 9.

**Table 3:** Parameter values of the model with a valve height of 14.5 mm.

**Parameter/D_**AA**_ (mm)**	**26**	**27**	**28**	**29**	**30**
Maximum stress (kPa)	639	701	576	741	793
Contact force (N)	8.46	7.03	7.55	7.71	6.66
GOA (mm^2^)	215.30	234.39	207.17	259.90	251.33

All the models with a valve height of 14 and 14.5 mm had complete closure and normal movement of the leaflets. The models with an H_V_ of 14.5 mm had smaller stress, larger GOA, and little change in the contact force compared with the models with an H_V_ of 14 mm (Figure 9). Thus, small maximum stress, reasonable leaflet contact force, and GOA have the smallest influence on the opening and closing performance of the aortic valve and are most consistent with the normal physiological state of the human body.

### Setting H_V_ to 15 mm

**Figure 10:**
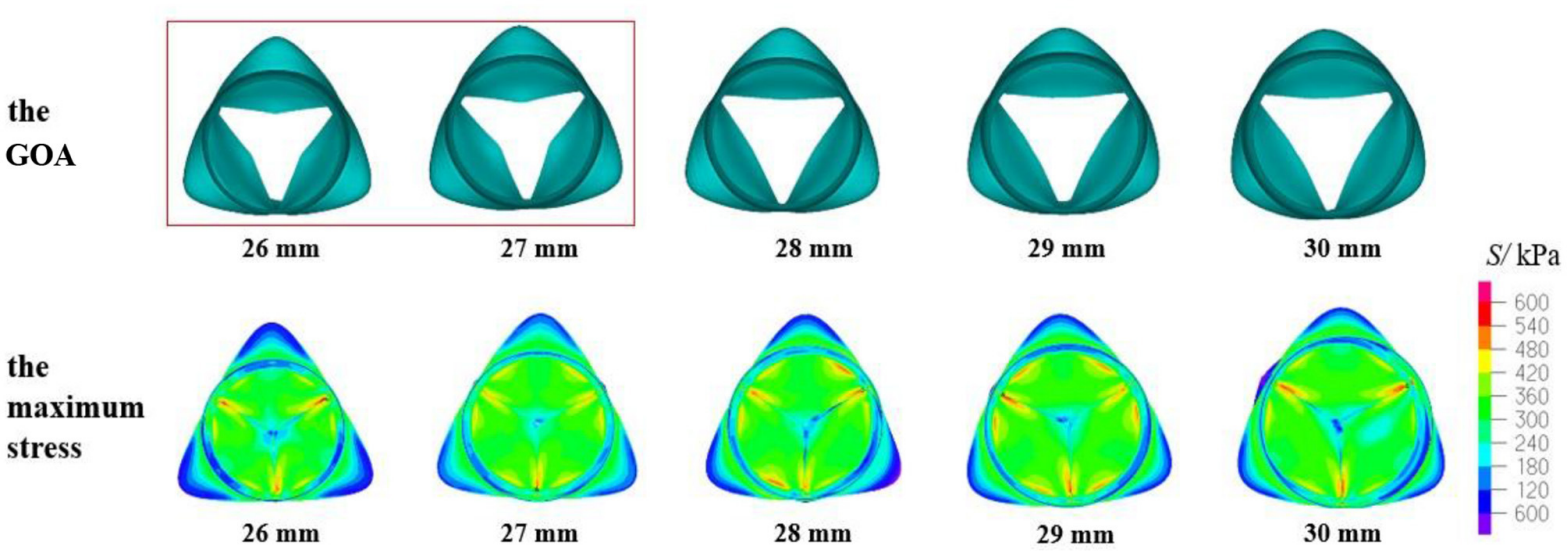
GOA of the valve and maximum stress distribution of the model with H_V_ = 15 mm and D_AA_ of 26–30 mm.

The mechanical parameters of the aortic valve with a valve height of 15 mm are shown in Table 4. Under the five values of D_AA_, the maximum stress and the leaflet contact force were within the normal range. However, according to the values of the GOA and the contact force (shown in Figure 10), the GOA was lower than the standard value, and the contact forces were greater than the other models when the D_AA_ was 26 or 27 mm. When the D_AA_ was 28, 29, or 30 mm, an H_V_ value of 15 mm could be well adapted.

**Table 4:** Parameter values of the model with a valve height of 15 mm.

**Parameter/D_**AA**_(mm)**	**26**	**27**	**28**	**29**	**30**
Maximum stress (kPa)	677	653	672	603	569
Contact force (N)	9.01	8.56	7.34	7.01	6.32
GOA (mm^2^)	177.33	198.54	201.33	215.23	220.74

## Discussion

Aortic valve repair is an attractive alternative to prosthetic valve replacement in young patients with aortic insufficiency (Navarra et al., 2013; Emmanuel et al., 2016). The repair procedure has advantages of low occurrence rates of valve-related events and there is no need for lifelong anticoagulation therapy. However, the repaired valve may develop functional failure over time. Durability limitations become apparent by the end of the first postoperative decade, mainly because of progressive dilatation of the aortic root.

The three-dimensional geometry of a base aortic root was reconstructed using the geometric constraints and modeling dimensions suggested by Labrosse et al. (2011). Then, the diameters of the aortic annulus and the height of the aortic valve were modified to create 20 geometric models with different dimensions. In this study, we used the structural mechanics simulation method to simulate the process of aortic valve movement and simulated the dynamics of the aortic root over two cardiac cycles. Next, we used the data from the last cardiac cycle for the analysis. The performance of the aortic valve was quantified in terms of maximum stress, GOA, and leaflet contact force.

### Analysis of Different Models Affecting Maximum Stress

The maximum stress value is an important index to evaluate the mechanical performance of the valve. From a physiological aspect, excessive stress may tear the valve or accelerate the calcification process of the aortic valve because of the accumulation of inactive cells (Cao and Sucosky, 2017; Jia et al., 2017), thereby further aggravating aortic stenosis. Therefore, the value and distribution of the maximum stress during valve closure are of great importance to clinicians and researchers. The results of this study showed that the maximum stress presented a trend of weakly decreasing first and then sharply increasing in the cardiac cycle, bearing the highest stress during diastole. A previous study (Labrosse et al., 2010) has shown the maximum stress value for the aortic valve to be 600–750 kPa during the diastolic period. Qiao et al. used 3D structural models to study the influence of the sinotubular junction and sinus diameter on the valve. They reported the calculated maximum stress range of the model with normal motion in numerical simulation to be 567–601 kPa (Qiao et al., 2014). Marom studied the effect of D_AA_ on the valve and calculated the maximum stress to be 800 kPa (Marom et al., 2013). The results in this study showed that the maximum stress was generally distributed at the junction of the aortic valve and sinus. All the AI models had maximum stress values >800 kPa, and the degree of insufficiency became more serious as the stress increased. Therefore, the smaller the stress on the valve leaflet, the more conducive the efforts to maintain the long-term effectivity of the aortic valve under the premise of ensuring its normal function.

### Analysis of Different Models Affecting the Leaflet Contact Force

Leaflet contact force refers to the interaction force between leaflets. It could be indicative of the holding strength between leaflets during closing phases of the cardiac cycle (Marom et al., 2013; Pan et al., 2015). Dynamic leaflet contact force data generated during the simulation were saved to a file to perform further processing using the MATLAB environment. This was done to determine the surface area of coaptation at the time of maximum downward movement of the leaflets during diastole (Labrosse et al., 2011).

The simulation results show that just after initial leaflet separation, the pressure was ramped back down to 0 kPa with the contact management turned on, which allowed the leaflets to contact each other in a realistic fashion. The pressure was then raised to 13 kPa. The contact force applied on the coaptation area was not a parameter that exhibited large variations, ranging from 0.5 to 9 N (Tables 1–4). This pressure was calculated during diastole at the time when the pressure differential between the aortic and ventricular sides was 13 kPa. It makes intuitive sense that the higher the leaflet contact force, the better the valve coaptation. However, in general, this parameter does not appear to be a good predictor of coaptation quality. Excessive contact force can increase the energy consumption of leaflets, increase the risk of AR, and reduce the durability of the aortic valve. In this study, the contact force in the model in which the valve can be normally closed was >3 N during the whole cardiac cycle. This phenomenon is similar to the results previously reported by Li et al. (2019). In the model where the leaflet contact force was <3 N, the valve was incompletely closed. As a result, the contact between the leaflets may be abnormal if the contact force is too large or too small.

### Analysis of Different Models Affecting the GOA of Leaflet

Geometric orifice area is the critical reference index to evaluate the opening and closing characteristics of the aortic valve, the larger the opening area, the better the opening effect of the heart valve. First, we needed to determine the moment when the GOA has the largest value, and then the post-processor of ADINA was used to take top view snapshots of the valve at that moment during the cardiac cycle by projecting the valve on the virtual valvular ring plane. Lastly, we drew the space surface of the opening area according to the projected area and calculated its surface area using the SolidWorks 15.0 software.

According to the criteria of clinical diagnosis, the aortic valve orifice area could be defined according to three states: when the geometric orifice area is <200 and more than 150 mm^2^, it is mild stenosis; when the geometric orifice area is <150 and more than 100 mm^2^, it is moderate stenosis; when the geometric orifice area is <100 mm^2^, it is severe stenosis. Therefore, the clinical standard value for normal GOA is >200 mm^2^ (Garcia et al., 2011). The aortic valve orifice area with the valve in the fully open position for the TAV is shown in Figures 6, 7, 8, 10. The open area shown in these figures is more like a triangle but not a circle. This phenomenon is related with the geometry and material of the prosthetic valves. The demonstrated scenario is similar to that reported in Labrosse et al. and others (Kim et al., 2007, 2008; Jermihov et al., 2011; Labrosse et al., 2011; Hsu et al., 2014; Gilmanov and Sotiropoulos, 2016). Previous studies (Labrosse et al., 2011; Li et al., 2019) have calculated the maximum orifice area of all aortic root models with a normal valve function to be 210 ± 10 mm^2^, which compared well with *in vivo* data obtained from transesophageal echocardiography in 19 normal aortic valves with a maximum GOA of 270 ± 63 mm^2^. This is close to the calculated results in this article.

The results of this study show that only in two models (H_V_ = 15 mm, D_AA_ = 26, 27 mm) the geometric orifice area was <200 mm^2^, and that it was more than 200 mm^2^ in the other models, which met the standard value. However, when the GOA is <200 mm^2^, the aortic valve may suffer from stenosis, which leads to the obstruction of the left ventricular blood output and an increase in the afterload. The heart will, in fact, increase its own work to improve the cardiac output to satisfy the blood supply needs of the body, which is called the Starling compensation mechanism. Therefore, from the long-term development of the disease, this will increase the burden of the heart, which may cause serious heart diseases, such as myocardial thickening, cardiac hypertrophy, and other serious heart diseases.

### Limitations and Future Study

In this study, several assumptions on the model settings and materials were made to reduce complexity. The main limitations of this study include the following aspects: first, parameterized ideal models based on physiological anatomy were used instead of a patient-specific model. Although patient-specific models could actually reflect the morphology and structure of diseased valves and aortic roots, morphological differences between individuals were huge and sizes varied (Cao and Sucosky, 2015), making it difficult to quantitatively compare the differences between aortic roots. Parameterized models have been used to study the normal aortic valve function (Labrosse et al., 2011; Weltert et al., 2013; Pan et al., 2015; Halevi et al., 2016) and surgical repair techniques (Labrosse et al., 2011; Hammer et al., 2015). For these studies, the primary utility of computational methods is the ability to isolate and quantify the effect of single variables, such as size, shape, and mechanical properties of a given structure on measurable outcomes. In order to highlight the structural differences between different aortic roots and reduce the influence of other factors in the models, parameterized models were adopted in this study. Based on the initial model, the root dilatation and valve alterations in surgery were simulated by changing the annulus diameter and valve height, respectively, while the dimensions of other components, such as sinus diameter and valve height, remained unchanged. Among them, the three aortic sinuses and leaflets were assumed to be uniform and symmetrical, and the left and right coronary arteries were not considered in the sinus model, which was somewhat different from the physiological anatomy. The influence of the coronary artery structure on aortic valve closure function needs to be further discussed in the next study. Second, in this simulation study, the material of the aortic valve was assumed to have isotropic and linear elastic material properties to simplify the computation and improve the feasibility of the analysis and avoid the problem of excessive distortion of the mesh during the contact process of the leaflets. In fact, the aortic valve tissue exhibits an obvious fiber arrangement (Feng et al., 2020), which is a characteristic of a hyperelastic and anisotropic material. Studies have shown that isotropic and anisotropic leaflet materials are not expected to significantly affect the dynamic performance of leaflets (Hammer et al., 2016; Cao and Sucosky, 2017). However, in order to study the mechanical properties of the valve, including stress and strain on leaflets, it may be necessary to consider the anisotropic constitutive mode in future studies.

Finally, under normal physiological conditions of the human body, the aortic root is in the flow field. When the blood flow from the left ventricle acts on aortic root components, such as the leaflets and sinus, stress distribution is different in each part. However, the idealized model of the aortic root constructed in this study did not consider the influence of blood flow, and the pressure load was uniformly applied to the valve and the vessel wall. Nevertheless, in the fluid-structure interaction (FSI) simulation study (Marom et al., 2016), the movement of the commissures was found to be somewhat small (<2%). This simplification is not expected to significantly affect the kinematics of the leaflets, and therefore should have a great influence on hemodynamic and wall shear stress (WSS) predictions. The purpose of this study was mainly to focus on the mechanical properties and structural changes of the leaflets. Some studies have pointed out that structural simulations are forced to assume spatially uniform transvalvular pressure (Haj-Ali et al., 2008; Conti et al., 2010a,b), which was found to be higher than the pressure load on the leaflets. As the leaflet tissue is soft, this pressure load is enough to deform the leaflet. The larger pressure load yields larger coaptation area, contact force, and stress in the structural model. Therefore, the use of structural models is justified for stress analysis, durability, or calcification evaluation, since the larger predicted stress gives a conservative estimate. In future studies, fluid-structure coupling analysis and pulsating flow experiments *in vitro* can be performed to obtain a more comprehensive and accurate conclusion.

## Conclusion

This study has shown that aortic annulus diameter and valve height are important factors influencing the performance of aortic valve closure, especially with regard to valve repair operation. The results show that within the 20 designed models, the aortic valve geometry with an H_V_ of 13.5 mm is suitable for the small aortic root geometry, and that best choices for D_AA_ are 26, 27, or 28 mm. An H_V_ of 14 or 14.5 mm can well adapt to the five selected D_AA_. Compared with the model with an H_V_ of 14 mm, the model with an H_V_ of 14.5 mm has relatively small stress, large GOA, and little change in the contact force. The aortic valve with an H_V_ of 15 mm is suitable for the larger aortic root and better choices for D_AA_ are 28, 29, or 30 mm. Hence, a smaller H_V_ is adapted to a smaller D_AA_ and vice versa. When H_V_ is 14.5 mm, the mechanical performance of the valve is good and can well adapt to the dilatation of the aortic root to enhance repair durability. All these findings suggest that more attention should be paid to H_V_ during surgical planning.

## Data Availability Statement

The original contributions presented in the study are included in the article/supplementary material, further inquiries can be directed to the corresponding author/s.

## Author Contributions

QH was responsible for modeling, simulation, data analysis, and article preparation. GL assisted in data analysis. HL assisted in the design of numerical simulation. NL, HoZ, ZQ, and HaZ were responsible for language modification. YP assisted in the construction of the 3D model. AQ was responsible for supervision. All authors contributed to the article and approved the submitted version.

## Conflict of Interest

The authors declare that the research was conducted in the absence of any commercial or financial relationships that could be construed as a potential conflict of interest.

## Publisher's Note

All claims expressed in this article are solely those of the authors and do not necessarily represent those of their affiliated organizations, or those of the publisher, the editors and the reviewers. Any product that may be evaluated in this article, or claim that may be made by its manufacturer, is not guaranteed or endorsed by the publisher.
